# Sex differences in physiological response to increased neuronal excitability in a knockin mouse model of pediatric epilepsy

**DOI:** 10.1042/CS20231572

**Published:** 2024-02-21

**Authors:** Michael F. Hammer, Collin T. Krzyzaniak, Erfan Bahramnejad, Kiran J. Smelser, Joshua B. Hack, Joseph C. Watkins, Patrick T. Ronaldson

**Affiliations:** 1 BIO5 Institute, University of Arizona, Tucson, Arizona, U.S.A.; 2 Department of Neurology, University of Arizona, Tucson, Arizona, U.S.A.; 3 Department of Pharmacology, University of Arizona, Tucson, Arizona, U.S.A.; 4 Department of Mathematics, University of Arizona, Tucson, Arizona, U.S.A.

**Keywords:** blood brain barrier, model organisms, Scn8a, sex dimporphism, transcriptomics, voltage-gated sodium channel

## Abstract

**Background**: Epilepsy is a common neurological disease; however, few if any of the currently marketed antiseizure medications prevent or cure epilepsy. Discovery of pathological processes in the early stages of epileptogenesis has been challenging given the common use of preclinical models that induce seizures in physiologically normal animals. Moreover, despite known sex dimorphism in neurological diseases, females are rarely included in preclinical epilepsy models.

**Methods**: We characterized sex differences in mice carrying a pathogenic knockin variant (p.N1768D) in the Scn8a gene that causes spontaneous tonic-clonic seizures (TCs) at ∼3 months of age and found that heterozygous females are more resilient than males in mortality and morbidity. To investigate the cellular mechanisms that underlie female resilience, we utilized blood–brain barrier (BBB) and hippocampal transcriptomic analyses in heterozygous mice before seizure onset (pre-TC) and in mice that experienced ∼20 TCs (post-TC).

**Results**: In the pre-TC latent phase, both sexes exhibited leaky BBB; however, patterns of gene expression were sexually dimorphic. Females exhibited enhanced oxidative phosphorylation and protein biogenesis, while males activated gliosis and CREB signaling. After seizure onset (chronic phase), females exhibited a metabolic switch to lipid metabolism, while males exhibited increased gliosis and BBB dysfunction and a strong activation of neuroinflammatory pathways.

**Conclusion**: The results underscore the central role of oxidative stress and BBB permeability in the early stages of epileptogenesis, as well as sex dimorphism in response to increasing neuronal hyperexcitability. Our results also highlight the need to include both sexes in preclinical studies to effectively translate results of drug efficacy studies.

## Introduction

Epilepsy is a common neurological disorder affecting 1–2% of the population worldwide. With young children and the elderly especially prone, there is a lifetime risk for developing epilepsy of 1 in 26 [1]. Epileptogenesis, the process through which neuronal networks are altered resulting in the generation of spontaneous, chronic seizures, is thought to involve three stages: (1) the initial insult, (2) the latent period, and (3) the chronic epilepsy phase [2]. The initiating event(s) leading to epilepsy may be genetic or acquired (e.g., through injury or disease) [3]. Despite decades of drug development, few if any of the currently marketed antiseizure medications prevent or cure epilepsy, and a large proportion of patients (at least one-third) are refractory to current treatments [1]. Preclinical research has focused on models in which seizures are induced by chemoconvulsants or electrical stimulation in physiologically normal or ‘naïve’ animals [4]. This approach potentially leaves gaps in our understanding of inherent pathophysiological processes that occur in the establishment of seizures and their subsequent propagation. Additionally, most preclinical studies in epilepsy have focused on males, impeding our understanding of the pathological processes that distinguish the sexes [1,5–7]. This is a serious oversight given the increasing evidence that sex differences in underlying brain function and in the neurobiology of epilepsy are important factors that should be accounted for in the design and development of new therapies [1]. Indeed, many neurodegenerative and neurodevelopmental disorders, including the epilepsies, appear to share a common pathogenic ‘triad’ that includes glutamate excitotoxicity, oxidative stress, and neuroinflammation— all of which show sex-dependent susceptibility patterns [4,8–12].

The increasing number of transgenic mouse models established over the last three decades offers the opportunity for careful and controlled investigation of sex differences in the initial phases of epileptogenesis and beyond [2]. Unfortunately, there have not been many systematic studies of sex differences with knock-in alleles associated with epilepsy [13,14]. We engage in such a systematic study by investigating a mouse model with a pathogenic gain of function (GOF) variant in the *SCN8A* gene, which encodes the Na_V_1.6 voltage-gated sodium channel. One of the most abundant sodium channels in the human brain, Na_V_1.6 channels regulate the initiation of action potentials and are important contributors to neural excitability. *SCN8A* GOF variants are associated with a wide spectrum of pediatric disorders ranging from benign familial seizures to severe developmental and epileptic encephalopathy (DEE) [15,16]. In 2015, a preclinical model was constructed with the index patient variant—N1768D—on the C57BL/6J background in which heterozygous (D/+) mice develop tonic-clonic seizures (TCs) between 2.5 and 3 months of age, exhibit ictal discharges coinciding with these seizures [17], and experience early death.

We previously characterized the natural history of Scn8a-D/+ mice of both sexes and found that females were more resilient than males in morbidity and mortality, experiencing more TCs yet surviving significantly longer [18]. These results led us to hypothesize that processes governing susceptibility or resilience in our novel model of epileptogenesis may be members of the aforementioned pathogenic ‘triad’. We also postulate that another pathogenic process that is known to be affected by sex hormones: blood–brain barrier (BBB) dysfunction, may be a key pathology in this model. Indeed, BBB permeability has been shown to be associated with epileptogenesis [19,20] and to exhibit sex dependent susceptibilities [21–23]. In the present study, we perform hippocampal transcriptome and pathway enrichment analysis in D/+ versus wild-type (+/+) mice of both sexes at 6 weeks and at ∼3.5 months to investigate alterations of cellular processes at time points before and after seizure onset (i.e., after experiencing ∼20 TCs), respectively. Additionally, we evaluate brain uptake of radiolabeled sucrose, a small molecule (MW 342.3 Da) tracer that does not typically cross the BBB under physiological conditions [24], at the same time points to test for sex-differences in BBB permeability in early and chronic stages of epileptogenesis.

## Materials and methods

### Animals and phenotyping

Female and male mice on the C57BL/6J (B6) background were housed in sex-specific groups of 3–4 per cage in a pathogen-free mouse facility with a 14 h light/10 h dark cycle (lights turned on at 5 am). Transparent polycarbonate cages were provided with bedding and a small amount of enrichment material to allow mice to be observable by camera mounted over the enclosure. All efforts were made to minimize animal stress and suffering and to reduce the number of mice used. Experiments received formal approval from the University of Arizona Institutional Animal Care and Use Committee Program (IACUC #16-160). All experiments were designed in accordance with the Animal Research: Reporting In Vivo Experiments (ARRIVE) guidelines [25]. Genotyping at the Scn8a-N1768D site to distinguish D/+ and +/+ mice was carried out as previously described [18]. A 24/7 video monitoring system was utilized to collect seizure data, with infrared illumination to monitor behavior during the dark period. Seizures were counted as individual TCs as described in [18].

### RNA sequencing

Hippocampal tissue from 24 mice were obtained according to a sampling strategy that compared both female and male D/+ pre-seizure mice (*n* = 3 per group), as well as D/+ mice that had experienced ∼20 TCs (*n* = 3 per group), with age-matched +/+ controls (*n* = 3 per group). Bulk tissue was stored in RNALater (Qiagen, Valencia, CA) at −80 degrees centigrade. The technique for analyzing hippocampal gene expression was performed as previously described [26,27]. Briefly, RNA was isolated from hippocampal tissue and initial QC performed. Libraries were constructed using a stranded mRNA-Seq Kit and average fragment size was assessed. After concentrations were determined with an adaptor-specific qPCR kit, equimolar samples were pooled and clustered for sequencing on a Novaseq instrument (Illumina). Sample data were demultiplexed, trimmed and quality filtered, and Fastq files were splice aligned against the GRCh37 reference genome using STAR aligner version 2.5.2b [28]. Gene expression counts were obtained using htseq-count version 0.6.1 [29]. Both splice alignment and counting were performed with Ensembl Annotation of the NCBI reference genome and raw counts analyzed with edgeR version 3.16.5 [30]

### Differential expression and pathway enrichment analysis

Differential expression analysis utilized the exactTest function in edgeR, which uses Benjamin–Hochberg correction to compute an upper bound for the expected false discovery rate (FDR). Gene expression counts were first normalized using the calcNormFactors function, which uses the trimmed mean of M values (TMM) to create a set of scaling factors that eliminates composition biases between sample libraries. Due to the variance between samples, the trended dispersion (the dispersion calculated from gene trancript abundance) was used for the exactTest calculation. All significant differentially expressed genes (DEGs) (FDR < 0.05) were analyzed with Ingenuity Pathway Analysis® (IPA) to identify biological pathways that were significantly activated or deactivated as compared with controls and to identify putative upstream transcriptional regulators (Qiagen, Hilden, Germany). We also utilized the Analysis Match function in IPA to identify closely related transcriptome datasets in the literature.

### Blood–brain barrier (BBB) analysis

Changes in BBB integrity were assessed by enhanced brain accumulation of ^14^C-sucrose (PerkinElmer Life and Analytical Sciences, Boston, MA). *In situ* perfusion with radiolabeled sucrose was performed as previously described [21,31–33]. Mice were anesthetized with ketamine/xylazine (K: 50 mg/kg; X: 10 mg/kg, i.p.) and heparinized (10,000 U/kg, i.p.) to ensure anticoagulation. An incision was made in the neck and the right carotid artery was exposed and cannulated. Following cannula placement, the mouse was perfused with an artificial plasma solution (i.e., 117 mM NaCl, 4.7 mM KCl, 0.8 mM MgSO_4_, 1.2 mM KH_2_PO_4_, 2.5 mM CaCl_2_, 10 mM d-glucose, 3.9% [w/v] dextran [molecular weight: 60,000], and 1.0 g/liter bovine serum albumin [type IV], pH 7.4, warmed to 37°C and continuously oxygenated with 95% O_2_/5% CO_2_) containing [14C]sucrose (0.5 mCi/ml; delivered via a slow-drive syringe pump) and both jugular veins were severed to allow drainage. Evan’s blue (55 mg/L) was also added to the perfusion buffer to enable a visual assessment of BBB integrity. Perfusion pressure and flow rate were maintained at 95–105 mmHg and 2.5 ml/min. After 10 min of perfusion, the cannulae was removed and the animal was decapitated. The brain was rapidly removed, the meninges and choroid plexus were excised, and cerebral hemispheres were sectioned. TS2 tissue solubilizer (1.0 ml; Research Products International, Mt. Prospect, IL) was added to the tissue samples, which were allowed to solubilize for 2 days at room temperature. To eliminate chemiluminescence, 100 μl og 30% glacial acetic acid was added, along with 2.0 ml of Optiphase SuperMix® liquid scintillation cocktail (PerkinElmer Life and Analytical Sciences). Radioactivity of [^14^C]sucrose was measured by liquid scintillation counting using a model 1450 Liquid Scintillation and Luminescence Counter (PerkinElmer Life and Analytical Sciences). Results were reported as picomoles of radiolabeled sucrose per milligram of brain tissue (RBr; pmol/mg tissue), which is equal to the total amount of ^14^C-sucrose in the brain (*R*_Brain_; dpm/mg tissue) divided by the amount of radioisotope in the perfusate (*R*_Perfusate_; dpm/pmol) (eqn 1):

(1) $$\text{RBr}=\frac{{\text{R}}_{\text{Brain}}}{{\text{R}}_{\text{Perfusate}}}$$

### Data analysis

Results of statistical summaries were generally expressed as mean ± SD. Kaplan–Meier survival curves were used to test for differences in survival. In cases where groups did not have the same variance, we performed two-sample *t*-tests. Chi-square tests were applied to test for sex differences in modes of deaths. Because many canonical pathways share common genes, we used a post-hoc method to aid in prioritizing pathways identified by IPA, which was helpful in cases where selected canonical pathways shared many redundant genes [34]. To do this we created a distance matrix based on 1-Jaccard coefficients [35] between pathways in each dataset and then constructed unrooted dendrograms based on the dissimilarity matrices. We also ran Fisher exact tests on shared *versus* unique genes in each pathway and made heat maps depicting significance of gene overlap among pathways.

## Results

### Sex differences in epilepsy resilience

**Figure 1:**
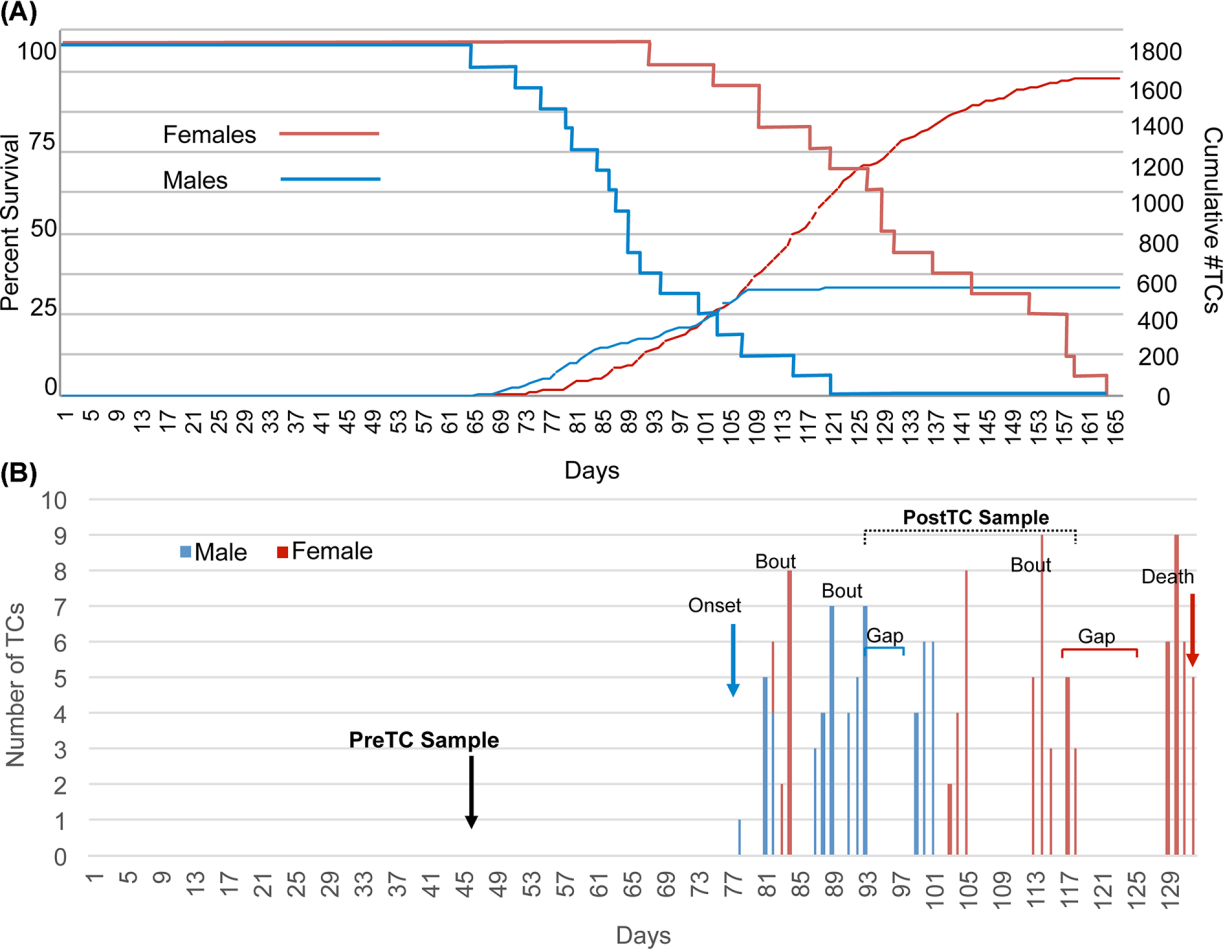
Lifetime number of TCs, survival, seizure bouts and gaps (**A**) Kaplan–Meier survival curves (solid lines) for 17 females and 17 males. Log rank test *P*-value <0.0001. Cumulative number of TCs is shown in dotted lines. (**B**) Pattern of TC frequency by day for a representative female (red vertical lines) and male (blue vertical lines) illustrating seizure onset, bouts, gaps and time of death. Time-points for sampling are indicated with bold arrow (pre-TC) or with bracket (post-TC).

Figure 1A displays Kaplan–Meier survival curves and cumulative number of TCs for 34 D/+ virgin females and males monitored 24/7 by video beginning at 30 days of age (P30) and continuing for the remainder of the life span. Twenty-four of the 34 mice were previously reported [18]. Females (*n*=17) had a cumulative total of 1563 TCs, while males (*n*=17) had a total of 538 TCs. All mice perished prematurely, with females living longer (mean: 132.9 ± 21.4 days; 95% CI: 122.7–143.1) than males (91.3 ± 15.2 days; 95% CI: 84.1–98.5) (*t*-test *P*-value <0.00001). Unequal survival was strongly supported in a goodness of fit test using the χ^2^ distribution (right-tailed) (*P*-value <0.00001), which also indicated a large observed standard effect size of 0.79. Age at seizure onset was earlier in males (76.2 ± 8.3 days; 95% CI: 72.2–80.1) than females (85.6 ± 13.8 days; 95% CI: 79.0–92.2) (*t*-test *P*-value = 0.011). Females had longer post-TC survival (46.6 ± 20.5 days; 95% CI: 36.9–56.4) than males (15.1 ± 14.0 days; 95% CI: 8.4–21.7) (*t*-test *P*-value <0.00001) and lower life-long seizure frequency (0.69 ± 0.23, 95% CI: 0.58–0.80 seizures/day *versus* 0.31 ± 0.30, 95% CI: 0.17–0.46 seizures/day) (*t*-test *P*-value = 0.00001) (Supplementary Table S1).

Figure 1B shows a typical seizure pattern (onset, seizures per day, seizure bouts and gaps without seizures for 3 or more days) in the life of a female and a male D/+ mouse. Adult females have more than a 3-fold greater number of gaps (3.7 ± 1.7; 95% CI: 2.9–4.5) than males (1.0 ± 1.1; 95% CI: 0.5–1.5) (*t*-test *P*-value< 0.00001), with gap lengths also significantly longer (6.6 ± 2.0 days, 95% CI: 5.6–7.5 *versus* 3.5 ± 4.5, 95% CI: 1.4–5.6, respectively) (*t*-test *P*-value = 0.008). The sampling scheme is also illustrated in Figure 1B, showing times of collection of pre-seizure (pre-TC) mice (P42-45) and collection after experiencing ∼20 TCs (post-TC) at approximately 90–110 days of age.

### Blood–brain barrier permeability before and after seizure onset

**Figure 2:**
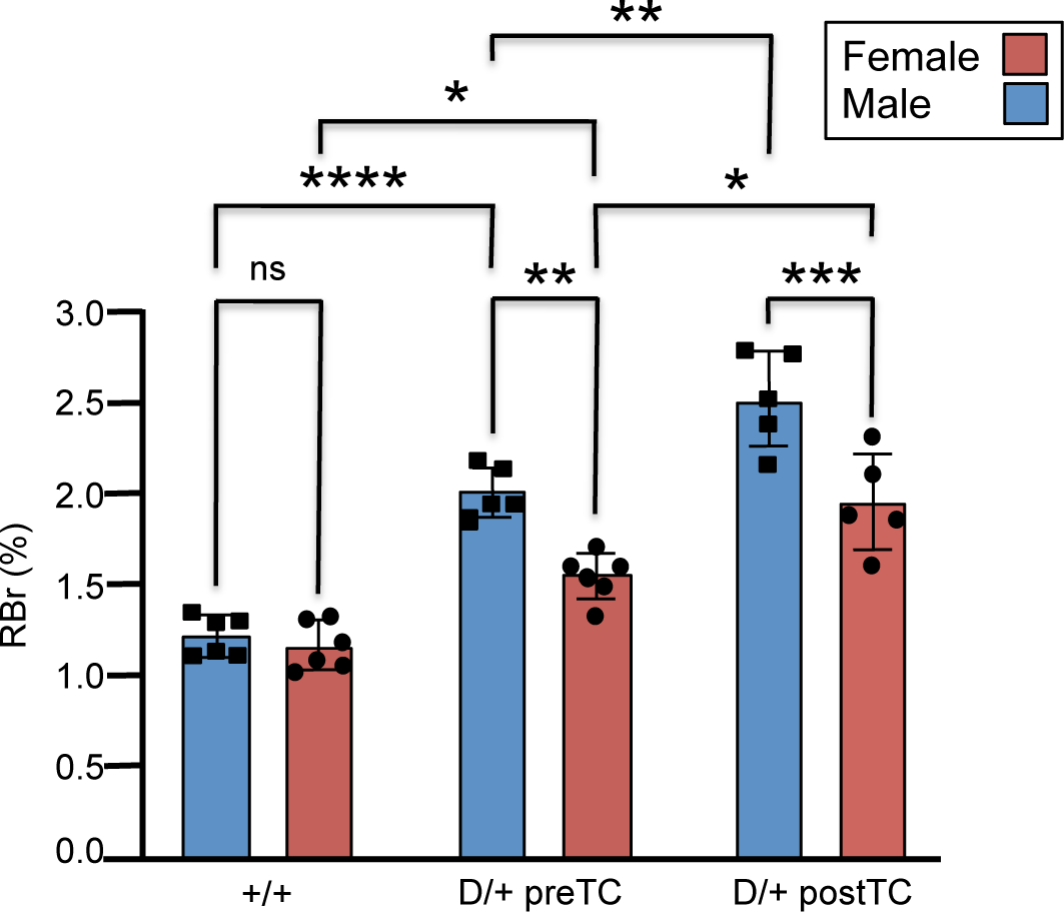
BBB paracellular permeability to sucrose is increased in male and female D/+ mice both before and after seizure onset *In situ* brain perfusion with 14C-sucrose as a vascular permeability marker shows significantly elevated radioactivity represented by brain-to-perfusate radioactivity ratios (RBr %) in brains of mice before and after detection of seizures and compared with controls (+/+ mice). Data are expressed as mean ± SD of 5–6 animals per treatment group (*P<0.05, **P<0.01, ***P<0.001, ****P<0.0001; ns = not significant).

To investigate the role of BBB function in epileptogenesis we performed *in situ* whole brain perfusion studies both before and after seizure onset. Our results show that BBB paracellular permeability (i.e., ‘leak’) to [^14^C]sucrose increased in both pre-TC D/+ females and males relative to wild-type controls (*t*-test *P*-value <0.05 and <0.0001, respectively) (Figure 2). The magnitude of the sucrose permeability increase that we measured in our knockin mouse model was greater in both post-TC D/+ females and males relative to pre-TC mice (*t*-test *P*-value <0.05 and <0.01, respectively). Wild-type females and males did not differ in permeability; however, both pre-TC and post-TC females exhibited permeability changes that were less than males at the same seizure stage (*t*-test *P*-value <0.01 and <0.001, respectively).

### Sexually dimorphic differential gene expression

**Figure 3:**
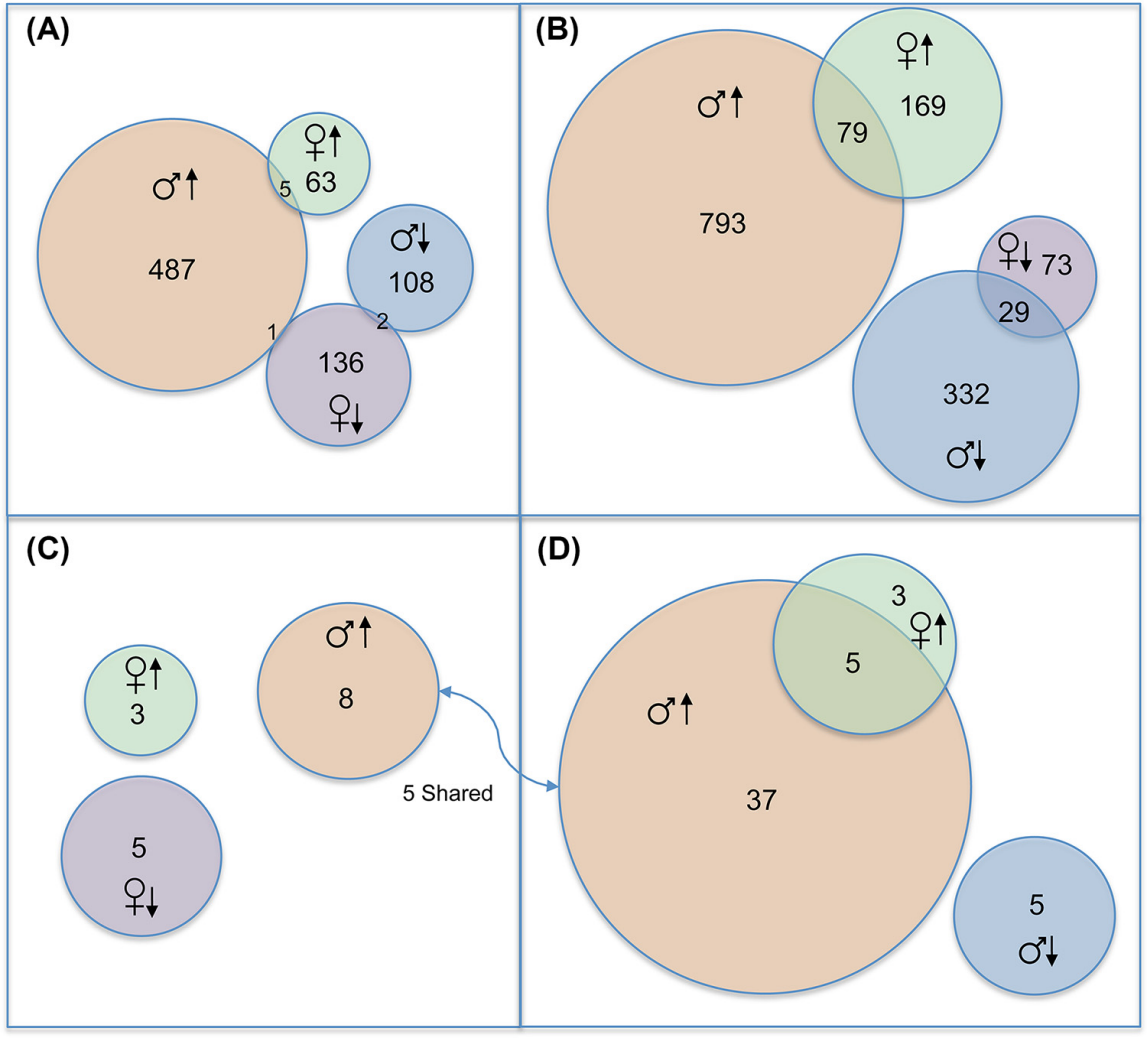
Genes and pathways identified by analysis of RNAseq data that are shared by the sexes or unique to each sex Number of differentially expressed genes (DEGs) identified in (**A**) pre-TC and (**B**) post-TC females and males. Number of significantly enriched canonical pathways identified by IPA in (**C**) pre-TC or (**D**) post-TC females and males. Female and male symbols and up or down arrows refer to number of genes or pathways that were up/down regulated or activated/deactivated, respectively.

To investigate cellular pathway alterations that may explain increasing BBB permeability, as well as those that may distinguish females from males in the pre- and post-TC stages, we performed RNAseq analyses on hippocampal samples. The experimental design shown in Figure 1B features a two-phase sampling strategy of D/+ females and males compared with +/+ (*n* = 3 per group) at 6-weeks pre-TC and after each D/+ mouse experienced ∼20 TCs (i.e., ∼P100). At the 6-week stage, there is a large sex difference in the number of differentially expressed genes (DEGs). Males had almost a 3-fold larger number of DEGs than females (of which 81.4% were upregulated *versus* only 32.8% in females) and there was very little sharing of DEGs and canonical pathways between the sexes (Figure 3A). Similarly, males had ∼3.5-fold more DEGs than females at the post-TC stage; however, there was a slight increase in the number of shared transcipts (∼7.3%) (Figure 3B).

### Canonical pathways in pre-TC females and males

**Figure 4:**
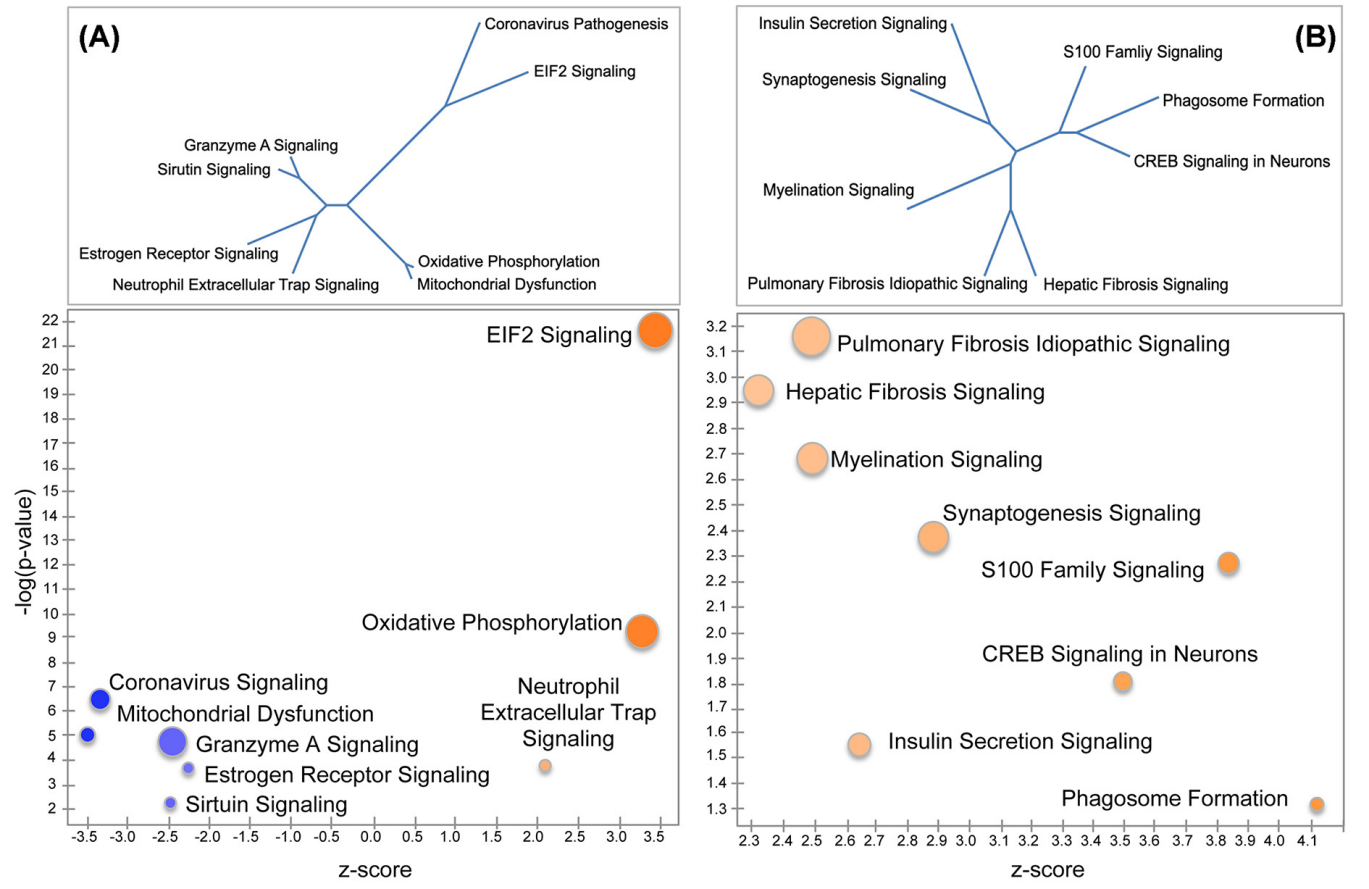
Canonical pathways in (A) pre-TC females and (B) males Bubble charts showing significantly enriched pathways (−log [*P*-value-] ≥ 1.3) that are color-coded by strength of activation (positive *z*-score ≥ +2.0) or deactivation (negative *z*-score ≤ −2.0). Circumference of pie chart represents ratio of number of overlapping genes (DEGs) to total number of genes in pathway. Dendrograms embedded within each bubble chart were constructed from distance matrices based on the Jaccard similarity index, calculated as the intersection over union for each pair of gene sets (see Materials and Methods).

Pathway enrichment analyses using IPA resulted in three significantly enriched canonical pathways predicted to be activated in pre-TC females and five that were predicted to be deactivated (Table 1 and Figure 3C). Males had a completely non-overlapping set of enriched pathways, all eight of which were inferred to be activated (Table 1 and Figure 3C). Several of these pathways were physiologically related and shared a subset of genes (e.g., pulmonary fibrosis idiopathic signaling pathway and hepatic fibrosis signaling pathway) (Figure 4A,B, top panels).

**Table 1:** Activated and deactivated canonical pathways in pre-TC females and males

	−log (*P*-value)	*z*-score
**Female**		
Activated		
EIF2 signaling	21.6	3.46
Oxidative phosphorylation	9.22	3.32
Neutrophil extracellular trap signaling	3.74	2.11
Deactivated		
Coronavirus signaling	6.47	-3.32
Mitochondrial dysfunction	5.02	-3.46
Granzyme A signaling	4.73	-2.45
Estrogen receptor signaling	3.64	-2.24
Sirtuin signaling	2.22	-2.45
**Male**		
Activated		
Pulmonary fibrosis idiopathic signaling	3.16	2.50
Hepatic fibrosis signaling	2.95	2.32
Myelination signaling pathway	2.68	2.50
Synaptogenesis signaling	2.37	2.89
S100 family signaling	2.27	3.84
CREB signaling in neurons	1.80	3.50
Insulin secretion signaling	1.55	2.65
Phagosome formation	1.32	4.12

Fifteen genes encoding subunits of the electron transport chain (ETC) complexes were up-regulated in females (Supplementary Figure S1). These included six within complex I (Ndufb6, Ndufb7, Ndufb9, Ndufa4, Ndufv3, and Ndufs6), two within complex II (Uqcrh and Uqcr10), four within complex IV (Cox5b, Cox6b1, Cox6c, and Cox7b), and three within complex V (Atp5k, Atp5j2, and Atpif1). In contrast, only six ETC transcripts reached a *P*-value ≤0.05 and none met the criterion of FDR ≤0.05 in males; however, all six genes were downregulated (Ndufb4, Uqcr10, Uqcr11, mt-Co2, mt-Co3, and Cox7c). Assuming a total of 96 ETC genes, the probability that all six would be down-regulated yields a *P*-value of 0.029 in a Fisher exact test.

To identify potential drivers of the differential expression pattern observed within each dataset we used the upstream regulator function in IPA. Of the biological molecules predicted to be activators in pre-TC females, a transcription regulator-MLX interacting protein-like (MLXIPL) was the molecule with the lowest *P*-value (2.11 × 10^−25^) and the most positive *z*-score (4.90). In pre-TC males, CTNNB1 and TCF7L2 were the top upstream transcription regulators (*P*-value = 2.27 × 10^−9^, *z*-score = 3.85, and *P*-value = 2.85 × 10^−13^, *z*-score = 3.57, respectively). β-Estradiol was also a top upstream activator (*P*-value = 2.17 × 10^−12^, *z*-score = 3.84).

### Canonical pathways in post-TC females and males

**Figure 5:**
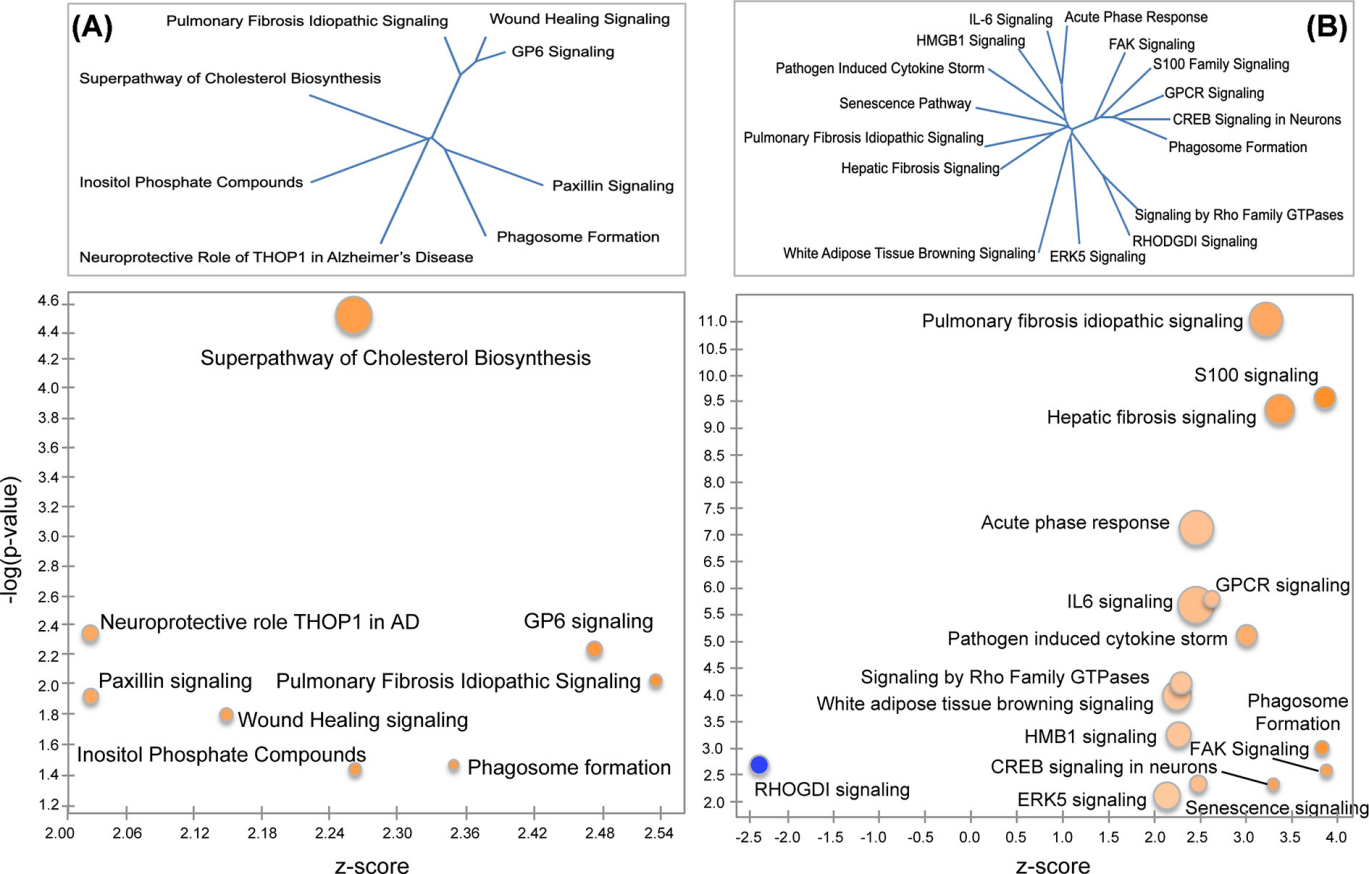
Canonical pathways in (A) post-TC females and (B) males See Figure 4 for the legend.

All eight significantly enriched canonical pathways in post-TC females were inferred to be activated (Table 2 and Figure 5A). In the case of post-TC males, 42 canonical pathways were predicted to be activated and 5 predicted to be deactivated (Table 2). The top 5 most significantly activated pathways utilizing an FDR of 0.05 included pulmonary fibrosis idiopathic signaling, hepatic fibrosis signaling, osteoarthritis pathway, STAT3 pathway, and wound healing signaling (Table 2). Figure 5B shows a reduced number of significantly enriched male post-TC canonical pathways with an FDR of 0.01.

**Table 2:** Activated and deactivated canonical pathways in post-TC females and males

	−log (*P*-value)	*z*-score
**Female**		
Activated		
Superpathway of cholesterol biosynthesis	4.52	2.24
Neuroprotective role of THOP1 in Alzheimer’s disease	2.33	2.00
GP6 signaling	2.23	2.45
Pulmonary fibrosis idiopathic signaling	2.03	2.53
Paxillin signaling	1.92	2.00
Wound healing signaling	1.80	2.12
Phagosome formation	1.47	2.32
Inositol phosphate compounds	1.45	2.20
**Male**		
Activated		
Pulmonary fibrosis idiopathic signaling	17.77	4.46
Hepatic fibrosis signaling	16.94	5.44
Osteoarthritis pathway	14.56	3.41
STAT3 pathway	13.26	2.45
Wound healing signaling	11.58	3.09
S100 family signaling	10.19	4.11
Human embryonic stem cell pluripotency	9.68	3.77
Role of JAK family kinases in IL-6-type cytokine signaling	9.08	2.24
Acute phase response signaling	8.78	2.68
Signaling by Rho family GTPases	8.52	2.79
TGF-β signaling	7.52	2.18
Phagosome formation	6.74	5.17
Actin nucleation by ARP-WASP complex	6.32	2.71
ILK signaling	6.26	2.56
G-protein coupled receptor signaling	6.23	2.90
IL-6 signaling	5.95	3.30
Actin cytoskeleton signaling	5.50	2.68
Inhibition of angiogenesis by TSP1	5.49	2.65
Pathogen-induced cytokine storm signaling	5.24	4.00
CREB signaling in neurons	5.11	4.53
Neuroinflammation signaling pathway	4.83	2.65
RAC signaling	4.43	2.89
Integrin signaling	4.38	3.55
FAK signaling	4.20	5.29
HIF1α signaling	4.08	2.86
IL-8 signaling	4.02	2.56
Regulation of actin-based motility by Rho	3.84	2.71
ERK5 signaling	3.65	2.33
PDGF signaling	3.54	2.31
BMP signaling	3.34	2.53
Paxillin signaling	3.16	2.12
Regulation of epithelial–mesenchymal transition	2.99	2.11
White adipose tissue browning	2.94	2.50
HMGB1 signaling	2.87	3.05
NF-κB activation by viruses	2.87	2.33
Ceramide signaling	2.82	2.53
IL-13 signaling	2.81	2.14
IL-15 production	2.57	2.50
LPS-stimulated MAPK signaling	2.08	2.12
GP6 signaling	2.04	2.50
Superpathway of inositol phosphate compounds	1.97	3.00
IL-3 signaling	1.84	2.12
Deactivated		
PTEN signaling	7.73	-2.52
RHOGDI signaling	6.97	-2.84
CLEAR signaling	4.58	-2.41
PPARα/RXRα activation	3.24	-2.67
PPAR signaling	3.16	-2.31

While there were no deactivated pathways shared between the sexes before seizure onset (Figure 3C), there were five activated pathways shared between post-TC females and post-TC males (Figure 3D). Four of these pathways were more significantly enriched in males and had higher activation scores (Supplementary Figure S2). There were five pathways shared between pre-TC and post-TC males, all of which were more significantly enriched and had higher activation scores after seizure onset (Supplementary Figure S2). This was particularly evident for the two fibrosis pathways (pre-TC vs. post-TC p-values: 6.9 × 10^−4^ vs. 1.70 × 10^−18^ and 1.12 × 10^−3^ vs. 1.14 × 10^−17^, respectively).

Of the biological molecules predicted to be upstream activators in post-TC mice, β-estradiol was the endogenous molecule with the lowest *P*-value (1.79 × 10^−12^) and the most positive *z*-score (5.93) in post-TC females. In post-TC males, TNF and TGFβ1 were the top predicted upstream activators (*P*-value = 1.68 × 10^−65^, *z*-score 7.34 and *P*-value = 1.54 × 10^−60^, *z*-score = 9.85, respectively). β-Estradiol and LPS were also predicted as top upstream activators (*P*-value = 3.26 × 10^−58^, *z*-score = 6.06; *P*-value = 5.97 × 10^−53^, *z*-score = 9.66, respectively).

## Discussion

**Figure 6:**
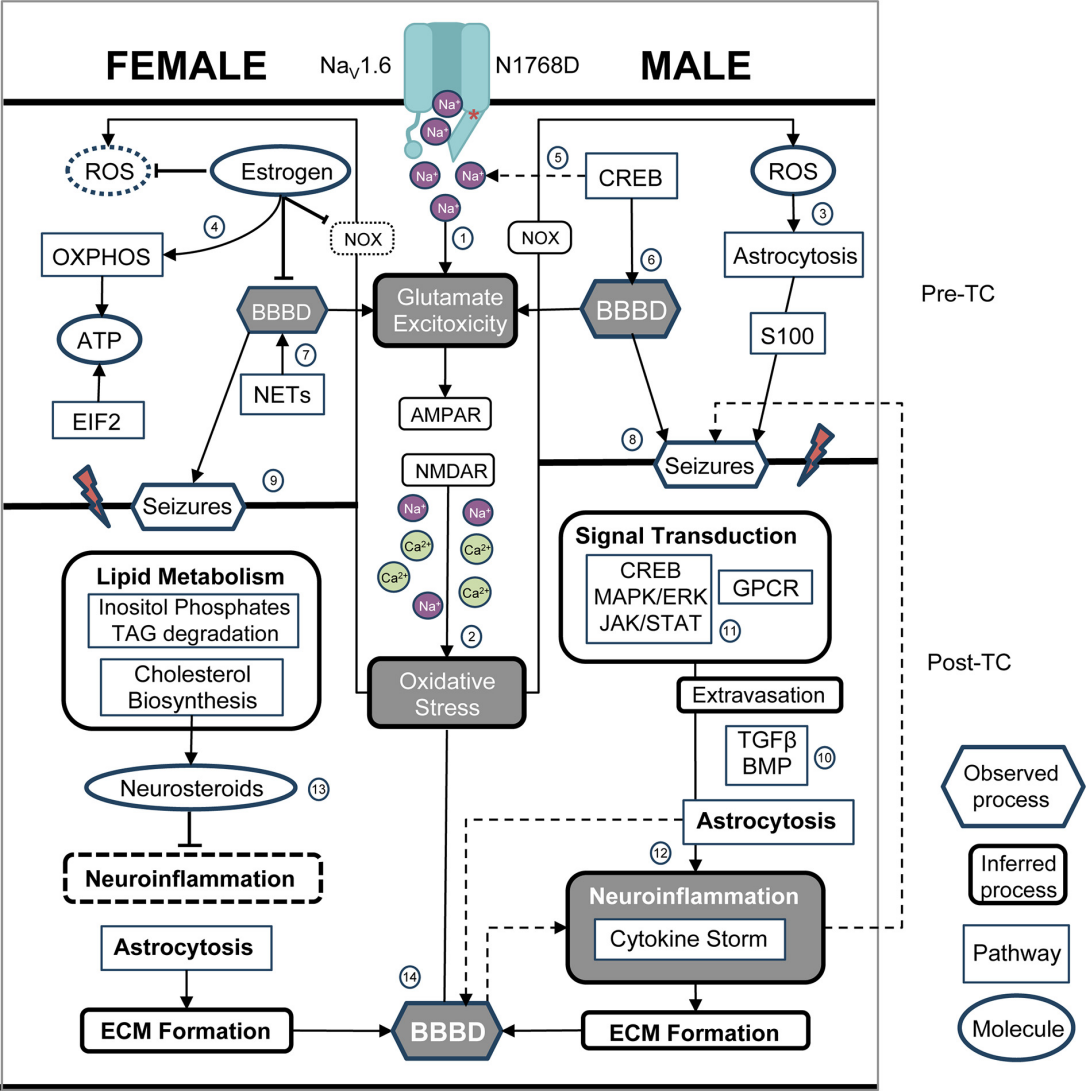
Integrative model for sex differences in epileptogenesis Impaired inactivation gating caused by the N1768D Na_V_1.6 variant is shown at the top, resulting in persistent sodium current, (1) glutamate excitotoxicity, and (2) downstream oxidative stress (OS). This process continues throughout pre-TC and post-TC stages, as shown centrally above and below the seizure onset lines, respectively. Reactive oxygen species (ROS) shown as potential inducers of (3) astrogliosis in pre-TC males, whereas (4) estrogen may in part mitigate effects of OS and extent of BBBD in females (see text), as well as influence increased protein biogenesis and OXPHOS. CREB signaling in neurons may have a direct effect in further enhancing (5) neuronal excitability and (6) BBB permeability (see text), whereas in females BBBD may be caused by (7) NET signaling. BBBD may lead to seizure onset (higher line in chart for (8) males than (9) females), marking the transition to more pathological conditions. In post-TC males, albumin extravasation via TGFβ/BMP signaling increases astrogliosis (10) and increased (11) MAPK/ERK/CREB signaling leads to activation of (12) neuroinflammatory pathways both of which increase (13) BBB permeability. Similar signaling in post-TC females is (13) suppressed (see text). Cytoskeletal remodeling associated with increased (13) BBBD occurs in both sexes. Activities that are part of the ‘tetrad of pathogenic processes’ common to neurological diseases (see text) are indicated in dark gray filled shapes. Feedback loops are indicated in arrows with dotted lines. Hexagonals, observed processes; rounded rectangles, inferred processes; rectangle with square corners, identified canonical pathways; ovals, inferred molecules.

We performed whole brain BBB and hippocampal transcriptome analyses on female and male samples to identify sex differences in epileptogenesis, as well as to discover pathway alterations that may explain increased female *versus* male resilience to morbidity and mortality in the Scn8a-N1768D mouse model of pediatric epilepsy. This model provides the opportunity to characterize the dynamic processes that occur naturally in response to GOF variants that lead to seizures— both in the period before seizure onset and in the chronic phase after seizures are established. Given that Na_V_1.6 channels play a major role in the initiation and propagation of action potentials and that N1768D Na_V_1.6 channels exhibit impaired inactivation and increased sodium flux in excitatory neurons, this model represents a classic case of increased excitatory neurotransmission induced by the release of excess glutamate, N-methyl-D-aspartate receptor (NMDAR) over-activation, and excess Ca^2+^ influx, triggering a cascade of events that includes mitochondrial dysfunction, oxidative stress, increased BBB dysfunction (BBBD), and neuroinflammation [36–40] (Figure 6).

This study is among the first to examine sex differences in the transcriptome of mice experiencing a natural onset of seizures [38,41]. Nearly 100% of heterozygous Scn8a^N1768D+/−^ (D/+) mice exhibited onset of tonic-clonic seizures beginning at 2–3 months, after an apparently normal period of development [18]. As shown in Figure 1 and Supplementary Table S1, D/+ female mice have a later age at seizure onset and can tolerate a greater number of seizures over a longer lifespan.

Overall, our results support the hypothesis that increased permeability (i.e., ‘leak’) of the BBB plays a key role in the development of seizures, as well as in the chronic stage of epileptogenesis. While BBBD was evident in both sexes, the extent of BBB permeability was found to be more severe in males. In addition, our results demonstrate large-scale sex-specific gene expression changes in response to the increased sodium flux of N1768D Na_V_1.6 channels during epileptogenesis. In the following sections we discuss different ‘adaptive strategies’ employed by females and males in response to increasing neuronal excitability in the pre- and post-TC stages. In some cases pathway disruptions are exacerbated after seizure onset and in other cases, pre-TC pathway alterations are abandoned in exchange for alternative compensatory mechanisms.

### Where the sexes agree: pathways maintaining the integrity of the BBB

While there was no sharing of canonical pathways between the sexes before seizure onset, there was convergence on five of the same pathways in post-TC mice, several of which are involved in cerebrovascular function and cytoskeletal remodeling (Supplementary Figure S2). Pulmonary fibrosis idiopathic signaling, wound healing, and GP6 signaling pathways all contain many collagens and laminins that comprise the basement membrane component of the BBB [42]. GP6 Signaling protects vascular integrity and prevents bleeding by recruiting platelets to neutrophil-induced vascular breach during inflammatory response. Similarly, Paxillin Signaling plays an important role in the reorganization of the actin cytoskeleton and the assembly/disassembly of focal adhesions, triggered by the engagement of integrins with the extracellular matrix (ECM) [43]. These alterations may underly the observed increases in BBB permeability shared between the sexes after seizure onset. The finding of activated NET signaling in pre-TC females may be associated with the moderately increased permeability of the BBB (Figure 6, #7), as the release of NETs from activated neutrophils [56] can damage the integrity of the BBB and aggravate neurological diseases through activation of microglia cells and increased secretion of IL-1β [44–46].

The increased severity of BBBD in males and their decreased resilience in general may be associated with the activation of neuroinflammatory pathways that lead to further deterioration of the BBB (see below). Increasing data point to the neuroprotective role of estrogen (especially E2) in regulation of the BBB [47–49], which may help to explain the milder extent of BBB disruption in females (Figure 2). These effects include reduction of ROS-induced tight junction dysfunction, prevention of leukocyte extravasation, mitigation of inflammatory response through inhibition of NFkβ expression, and neuroprotective signaling via an estrogen-astrocyte-TGFβ1 pathway [12,47]. Ovariectomy in female rats and mice increases paracellular BBB permeability and leads to significantly higher levels of brain proinflammatory cytokines and chemokines, acute disruption of which were prevented by estradiol replacement [50]. Estrogen is also implicated as a potent modulator of astrocytes in the CNS [50]. Interestingly, β-estradiol was predicted to be the most significant upstream activator in post-TC females. Less is known about the effects of androgens on BBB permeability and current results are somewhat contradictory [51].

### Transcriptome-wide gene expression alterations in the latent period

The combined evidence from the transcriptome sheds light on the early epileptogenic processes that occur before the onset of seizures, and that may represent physiological responses to increased neuronal excitability (Figure 6, #6). Changes in ETC gene expression in the Oxidative Phosphorylation (OXPHOS) (Figure 4A) pathway are associated with cellular stress in a number of neurological conditions [52,53]. Such alterations may represent an attempt by cells to increase their aerobic set point, or an attempt to maintain a pre-existing aerobic set point in the face of declining mitochondrial function [53]. Indeed, it is quite plausible that the increase in OXPHOS gene expression in pre-TC females represents a critical biological response to ATP depletion caused by Ca^2+^ overload after exposure of neurons to excess glutamate [40,54,55]. Interestingly, the gene (Mlxipl) encoding MondoA was predicted as the most significant upstream activator, a glucose-sensitive transcription factor that regulates genes that control complementary aspects of energy metabolism [56].

EIF2 signaling (Figure 4A) is often involved in the integrated stress response (ISR) [57,58], which has been observed in several models of epileptogenesis [59,60]. Typically, genes on this pathway are down-regulated in response to cellular stress [61,62]; however, in our dataset 23 of the 24 DEGs on the EIF2 signaling pathway were up-regulated, similar to results reported in a proteomic study of hippocampal tissue from epileptic adults [63]. One explanation for a coordinate expression of the OXPHOS and EIF2 signaling pathways is that augmented translation of ETC genes depends on increased expression of ribosomal proteins [64–66]. Interestingly, our results mirror those in a transcriptome study of pituitary samples from mice that were exercise-trained for three weeks compared with mice kept in a sedentary condition [67]. The coordinated activation of the EIF2 signaling and OXPHOS pathways in the trained mice was interpreted in terms of mitohormesis— the process by which moderate levels of mitochondrial stress lead to beneficial outcomes, with ROS being an essential requirement for this adaptation [53,68]. Indeed, the ETC complexes I, III, IV and V were increased to a similar magnitude in both studies (Supplementary Figure S1).

The two fibrosis pathways activated in pre-TC males (Table 1 and Figure 4B) are known to play a role in scar formation and tissue repair after injury. In the context of the CNS, this generally involves the activation of astrocytes [69]. The tight coupling between astrocytes and neurons via gap junctions facilitates rapid signaling responses during hyperactivity, and glutamate released from astrocytes can induce neuronal paroxysmal depolarizations that precede neuronal hyperactivity [70]— processes that may be ongoing in pre-TC males (Figure 6, #3). This is supported by the finding of activated S100 Family Signaling (Figure 4B and Table 2), proteins known to function in stress responses and a variety of Ca^2+^-dependent intracellular functions [71]. Similarly, our pre-TC male transcriptome analysis implicate activation of cAMP-responsive element binding protein (CREB) signaling in neurons (Figure 4B), which is often seen in association with recurrent epileptiform discharges or interictal spiking rather than with seizures themselves [72–74]. Chronic persistent Na^+^ current is expected to trigger excess glutamate release and activation of synaptic NMDAR, which has been shown to induce strong phosphorylation of CREB and promote CREB-dependent gene transcription [75]. In addition, NMDAR mediated hyperexcitability can lead to epileptiform activity and BBB disturbance early in the process of seizure development [73,76]. CREB-dependent transcription also may be upstream of the induction of synaptogenesis signaling [73,77] and myelination signaling [78,79], two additional activated pathways in pre-TC males. Ctnnb1 and Tcf7l2 were predicted as the most significant upstream activators in pre-TC males. Ctnnb1 encodes β-catenin, a key protein of the complex that mediates intercellular adhesion and an important member of the Wnt signaling pathway [80]. Tcf7l2 is also an important transcription factor in the Wnt pathway and is under tight regulation during myelin formation [81].

### Shift from oxidative metabolism in post-TC females

The activation of the Superpathway of Cholesterol Biosynthesis and an apparent switch from glucose to lipid metabolism are the main features of the post-TC transcriptomic signature in females (Figure 6). The increased dependency of pre-TC females on oxidative energy substrates for anabolism and energy production may represent an initial adaptive response to oxidative stress, yet it is unlikely that this strategy would continue to be beneficial over the long-term. Excess oxidation can eventually provoke metabolic failure, compromising cell viability by inactivating enzymes of glycolysis, the Krebs cycle, and even of the ETC [82]. While rates of glycolysis and OXPHOS are well matched in the resting brain, neuronal stimulation can cause a Warburg-like transient dissociation between glycolysis and OXPHOS in response to increased energy demand [83]. Even though glycolysis produces a lower yield of ATP than OXPHOS, neurons may favor glycolysis over OXPHOS in order to promote a faster resupply of energy [83]. It is also possible that a shift away from OXPHOS in post-TC females represents a bioenergetic strategy that utilizes lactose as an energy substrate and the pentose phosphate pathway (PPP) to increase production of NADPH, which is critically important for generating the reducing power that fuels antioxidant systems and the recycling of oxidized glutathione [82].

One explanation for activation of the synthesis of cholesterol— the primary substrate for biosynthesis of sex hormones and neurosteroids (Figure 6, #13)— is compensation for cholesterol loss or imbalance resulting from high excitatory neurotransmission via NMDAR [75,84,85]. The induction of genes related to cholesterol biosynthesis also may be associated with increased membrane production by reactive astrocytes [86], a pathway that is also significantly enriched/activated in our post-TC females (Figure 6). Support for the hypothesis that post-TC transcriptional changes reflect metabolic reprogramming comes from evidence for the activation of pathways within the family of inositol phosphate compounds (Figure 5A). The network of soluble and membrane-associated inositol phosphates coordinates cellular responses to nutrient uptake and utilization, playing a key role in the maintenance of energy homeostasis and metabolic reprogramming under conditions of metabolic challenge. Molecular changes induced by myo-inositol treatment may counteract epileptogenesis [87] via mechanisms that include reduction in ROS synthesis, direct superoxide scavenging, and protection of NO signaling [88].

### Male transition from physiological to pathological signaling

Unlike in females, several pathways that were activated before seizure onset were found to be more significantly activated in post-TC males (Table 2; Supplementary Figure S2A and B). This may represent a shift from physiological to pathological conditions after seizures become chronic. Persistent activation of astrocytes leads to a decrease in glutamate clearance with a corresponding accumulation in the synaptic extracellular space, increasing the chance of neuronal excitotoxicity [89]. Like much of homeostatic signaling in the brain, the multi-faceted nature of CREB signaling carries with it a ‘double-edged sword’ [90]. Brain gene expression studies have shown that mild pathology leads to a protective program of CREB-dependent transcription, whereas persistent CREB activation is directly linked to increases in epileptic seizures [74]. The pattern of increased activation of phagosome formation in post-TC males may reflect a similar shift from physiological to pathological conditions. With increased intensity and duration of neuronal hyperexcitability, microglia become more reactive. Short-lasting inflammation can promote neuroprotection by suppressing production of proinflammatory cytokines and promoting tissue repair; whereas longer-lasting inflammation can lead to neurodegeneration, cognitive decline, seizures, and epilepsy [91].

Interestingly, it was recently demonstrated that MAPK/ERK activation in dorsal root ganglia promotes the expression and activation of CREB, which binds directly to the promoter region of Scn8a, leading to an increase in Scn8a transcription. The increased expression of Na_V_1.6 protein enhances neuronal excitability [92], thus providing a direct link between CREB signaling and Na_V_1.6-related neuronal hyperexcitability and forming a positive feedback loop that could lead to weakening resilience in D/+ males (Figure 6, #5).

### Male-specific post-TC neuroinflammatory response

One of the most important factors distinguishing the sexes is the nearly exclusive activation of pathways that mediate the neuroinflammatory response in post-TC males (Table 2), such as those related to cytokine receptor signaling, NFkβ signaling, and the sterile inflammatory response [93]. Epileptogenesis and inflammation form a pathological positive feedback loop [94] that can be further amplified by increased BBB permeability [95] (Figure 6). During states of inflammation and increased cytosolic Ca^2+^, RhoA activation drives actin nucleation induced from stress fibers resulting in increased BBBD [96]. Activation of the IL-6 pathway is implicated in the progression of epilepsy, neuroinflammation and BBBD [95]. The results also implicate TGFβ and BMP signaling, important for regulation of the BBB, along with other signal transduction pathways involved in neuroinflammation (i.e., MAPK-ERK and HIF1α signaling). Interestingly, TNF and TGFβ1 were predicted as the most significant upstream activators in post-TC males (Supplementary Figure S3). They are both pleiotropic cytokines that regulate neuroinflammatory responses and BBB function, respectively [97]. PDGF Signaling may reflect the activity of proinflammatory cytokines on pericytes, which then activate microglia and contribute to BBB integrity loss and neuroinflammation in epilepsy [98]. Our results also highlight the importance of G protein-coupled receptor (GPCR) signaling, well known to play an important role in the regulation of neuronal excitability and setting seizure threshold [99] (Table 2).

Post-TC males also show a pattern of deactivation of PPAR signaling that is shared with other epilepsy syndromes, such as human TLE [26]. PPAR signaling involves a group of nuclear receptors that control lipid and glucose metabolism and energy homeostasis [100]. PPARγ has been detected in neurons and glial cells [101], where it acts in part by decreasing expression and release of proinflammatory cytokines, protecting mitochondrial function, and reducing the activation of microglia [102].

### A common pathogenic ‘tetrad’

It was previously recognized that a common pathogenic triad that includes glutamate excitotoxicity, neuroinflammation, and oxidative stress characterizes the neurobiology of various brain disorders, such as Alzheimer’s disease (AD), Parkinson’s disease (PD), amyotrophic lateral sclerosis (ALS), Huntington’s disease (HD), stroke, post-traumatic epilepsy, and temporal lobe epilepsy (TLE) [4,8–12,103]. Our results lead us to propose the addition of BBB disruption to form a ‘tetrad’ of common pathophysiological processes that include epileptogenesis [104–107]. These results also add to the current debate on the causative relationships between epilepsy, neuroinflammation, and BBB disruption. There is evidence that changes in BBB integrity are the direct consequence of seizure activity [108], while other studies suggest that BBB failure leads to seizures [109]. Additionally, there is controversy on whether BBBD and associated neuroinflammatory factors precede or follow the onset of seizure development [110–113]. Of translational importance, our results suggest that disruptions in BBB integrity can occur before seizure onset and the significant activation of neuroinflammatory pathways.

This work underscores both the importance of oxidative stress in epileptogenesis and sex differences in response to its effects. Sexual steroid hormones could be a major factor underlying these differences as they play a key role in the regulation of redox homeostasis in the brain. While both females and males have estrogens and androgens, their concentrations differ between the sexes [114]. Under physiological conditions, females and males differ in both the production and decomposition of ROS [9,115]. Males have a higher leakage of superoxide anion at the mitochondrial ETC and higher activity of pro-oxidant enzymes, such as xanthine oxidase (XO) and NADPH-oxidase (NOX). In addition, they have a lower expression and activity of important antioxidant enzymes such as superoxide dismutase (SOD) and glutathione peroxidase (GPx). These differences result in higher accumulation of oxidative damage over time in DNA, proteins, and lipids in males, impairing proper cellular and tissue function [11].

### Challenges and limitations

We faced several challenges in our interpretation of the transcriptome data. One challenge was to determine which of the cellular alterations discovered at each stage of epileptogenesis is a beneficial physiological response to an initial stress, and which changes represent dysfunctional or pathological processes. Moreover, given the significant sex differences in resilience observed in our dataset, the second challenge was to distinguish processes that were neuroprotective in females from those that were more likely to be pathological in males. Our general assumptions were to view alterations at the 6-week stage to represent physiological responses, and those in the post-TC stage to be pathological. In addition, given that Na_V_1.6 is also expressed in the peripheral nervous system we cannot rule out that some of the observed sex differences in resilience are the result of non-CNS alterations or those that take place in regions of the brain outside of the hippocampus [6,116].

There were several limitations in the data that should be noted. The first is the relatively small sample size for each sex/genotype group at each stage. Second, we only sampled mice at a single time-point within a relatively long latent period. Dynamics of transcriptome changes may be relatively rapid so more sampling periods are needed. Third, bulk tissue RNAseq cannot determine which cell types (neurons, astrocytes, microglia, etc.) are contributing to the list of DEGs, and indeed may miss opposing gene expression changes in different cell types. Single-cell RNAseq in a future study should shed light on key issues of compartmentalized responses in the hippocampus during each stage of epileptogenesis. It should also be noted that contrasting abundance of RNA transcripts in two different groups to identify DEGs and to infer pathways on which these DEGs are enriched can provide mechanistic insights; however, such analyses are unable to distinguish between causes, consequences, or mere correlations between gene expression and disease phenotypes [117]. Finally, there are biases in the knowledgebases used for pathway enrichment. These biases along with the statistical methods used to call pathway hits during enrichment analysis can lead to (1) over-calling pathways with redundant genes [34] and (2) difficulties in interpreting the biological role particular pathways in epileptogenesis when they were discovered (and labeled) in other diseases. Therefore, we consider our pathway analysis in a hypothesis consistency framework and as a tool to generate new hypotheses for future testing.

## Clinical perspectives

- Epilepsy is a common disorder; however, the pathophysiology of epileptogenesis and the molecular mechanisms underlying sex differences in epilepsy remain unclear.
- We observed blood–brain barrier dysfunction in both sexes prior to seizure onset, which was exacerbated in males once seizures were established. The initial male response of gliosis and CREB signaling likely led to maladaptive effects after seizures, while females initially activated energy enhancing pathways and switched to a more neuroprotective metabolic program in response to increased neuronal excitability.
- These findings reveal epileptogenic processes shared between the sexes, as well as sexually dimorphic responses that lead to increased seizure resilience in females.

## Supplementary Material

Supplementary Figures S1-S3 and Table S1

## Data Availability

The data that support the findings of the present study are available from the corresponding author on reasonable request. Raw data of RNA sequencing have been submitted to the Figshare repository (https://portlandpress.figshare.com)

## Open Access

Open access for this article was enabled by the participation of University of Arizona in an all-inclusive *Read & Publish* agreement with Portland Press and the Biochemical Society under a transformative agreement with Individual.

## Abbreviations

+/+: wild-type
AD: Alzheimer’s disease
ALS: amyotrophic lateral sclerosis
ARRIVE: Animal Research: Reporting In Vivo Experiments
ATP: adenosine triphosphate
B6: C57BL/6J
BBB: blood–brain barrier
BBBD: blood–brain barrier dysfunction
BMP: bone morphogenetic protein
CNS: central nervous system
CREB: cAMP-responsive element binding protein
CSE: convulsive status epilepticus
D/+: heterozygous
DEC: decompensation
DEE: developmental and epileptic encephalopathy
DEG: differentially expressed gene
E2: 17-β-Estradiol
ECM: extracellular matrix
ERK: extracellular signal-regulated kinase
ETC: electron transport chain
FDR: false discovery rate
GOF: gain of function
GPCR: G protein-coupled receptor
GPx: glutathione peroxidase
HD: Huntington’s disease
IPA: Ingenuity® Pathway Analysis
MAPK: mitogen-activated protein kinase
MLXIPL: MLX interacting protein-like
NETs: neutrophil exracellular traps
NFkβ: nuclear factor kappa-light-chain-enhancer of activated B cells
NMDAR: N-methyl-D-aspartic acid receptor
NO: nitric oxide
NOX: nicotinamide adenine dinucleotide phosphate oxidase
OXPHOS: oxidative phosphorylation
PD: Parkinson’s disease
Post-TC: post-seizure
PPP: pentose phosphate pathway
pre-TC: pre-seizure
ROS: reactive oxygen species
SUDEP: sudden unexpected death in epilepsy
TCA: tricarboxylic acid cycle
TC: tonic-clonic seizure
TGF: transforming growth factor
TLE: temporal lobe epilepsy
TMM: trimmed mean of *M* values
TNF: tumor necrosis factor
