# Supplementary material for: Sex differences in physiological response to increased neuronal excitability in a knockin mouse model of pediatric epilepsy
Source: Clin Sci (Lond). 2024 Feb 21;138(4):205–23. doi: 10.1042/CS20231572 (PMC10881277; doi:10.1042/CS20231572)
Supplement: Supplementary Figures S1-S3 and Table S1 [file CS-2023-1572_supp.pdf]

Supplementary Material

Supplementary Figures

Figure S1. Electron transport chain complex DEGs in pre-TC females and Walz et al. (62) exercise-trained mice (DUC tr vs sed). Left panel shows the absolute number of DEGs identified in each electron transport chain (ETC) complex and right panel shows number of DEGs relative to number of genes in entire complex.

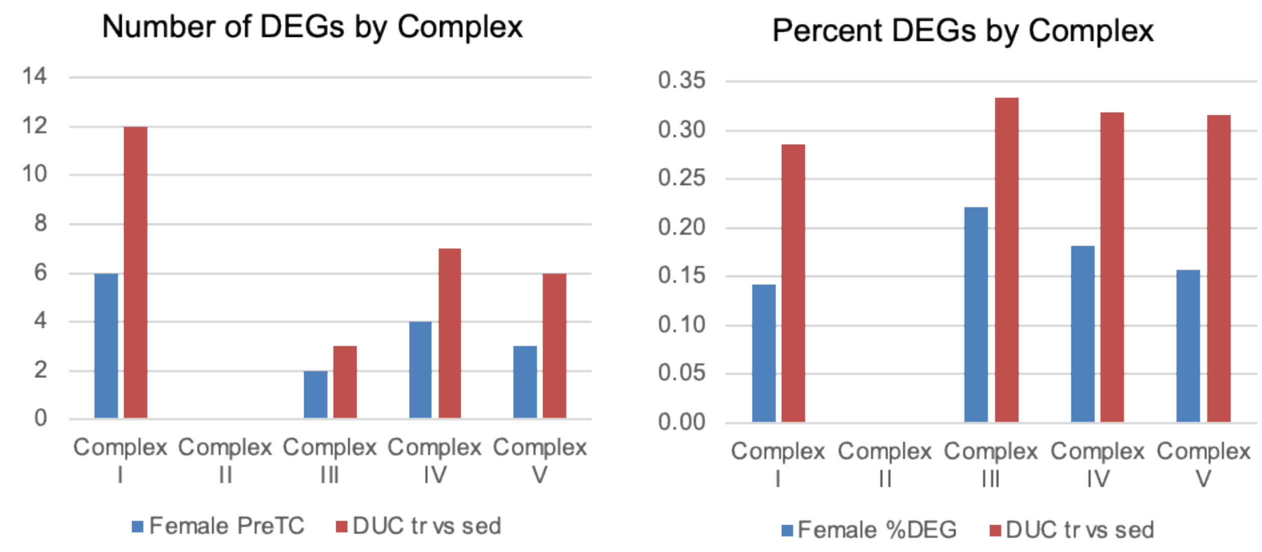

Figure S2. Shared canonical pathways between male pre-TC and post-TC (top) and between female and male post-TC (bottom). The left side shows the -log (p-value) associated with each significantly enriched pathway while the right side shows the z-score for each shared pathway (see text).

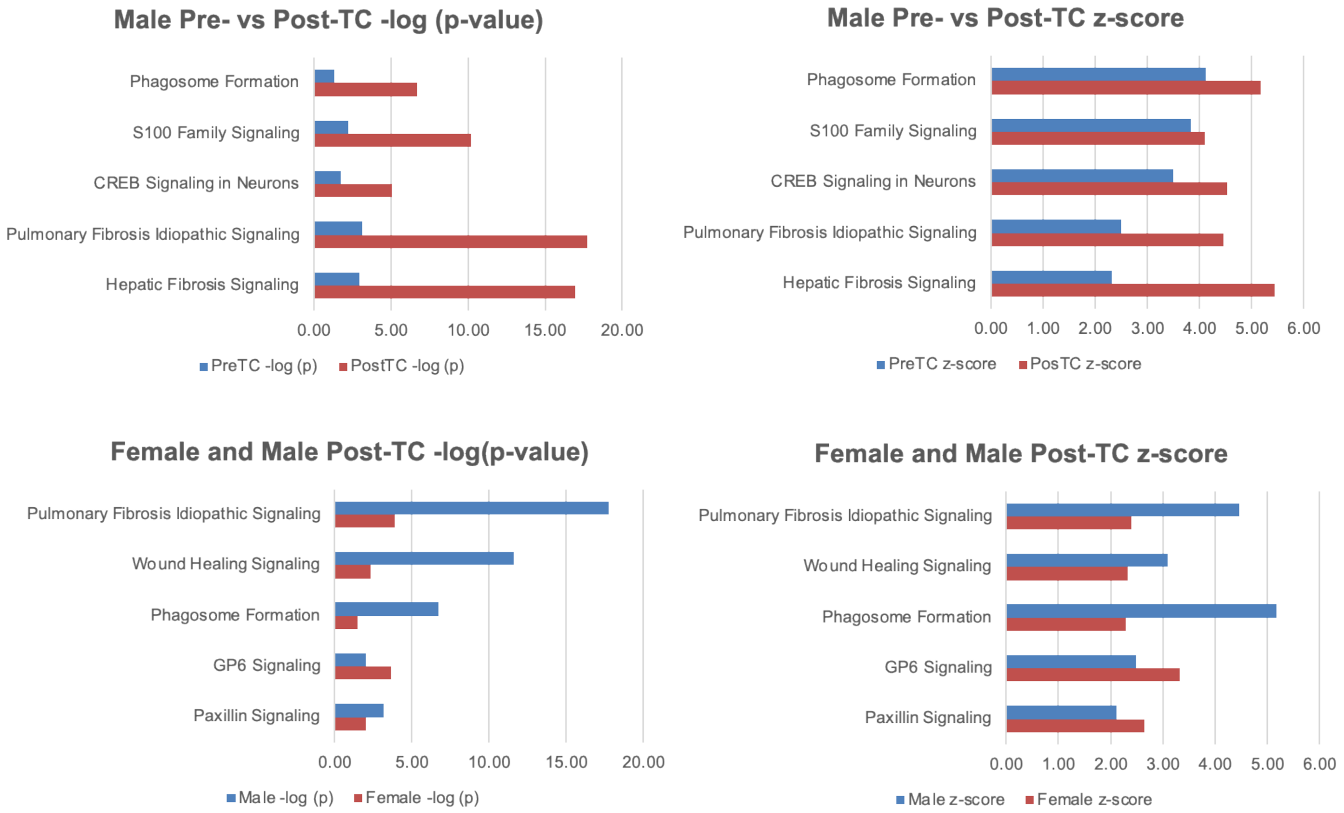

Figure S3. Upstream activator inferred for post-TC males by IPA (see methods).

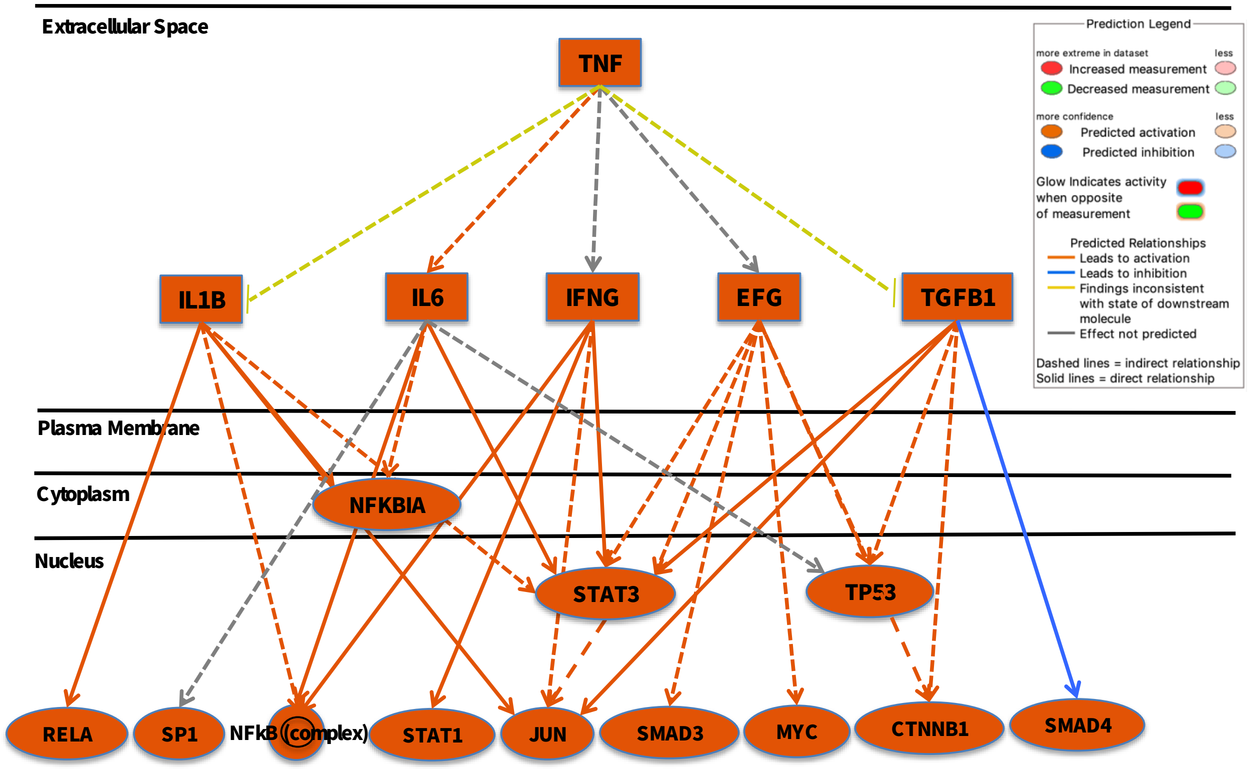

Table S1. Mortality and morbidity summary statistics

	Females	Males	p-value
sample size	17	17	na
Age at Death	132.9 (21.4)	91.3 (15.2)	<1 x10-5
#tonic-clonic (TC) seizures	91.9 (35.0)	31.6 (36.3)	1.2 x 10-5
Age at Onset	85.6 (13.8)	76.2 (8.3)	1.1 x 10-2
#Days survived postTC	46.6 (20.5)	15.1 (14.0)	<1 x10-5
#gaps (days)	3.7 (1.7)	1.0 (1.1)	<1 x10-5
mean gap length (days)	6.6 (2.0)	3.5 (4.5)	8.0 x 10-3
#gap days	24.3 (14.0)	4.8 (7.3)	1.2 x 10-5
MOD*	4,13,0	1,4,12	1.1 x 10-4

MOD, Mode of death (#SUDEP/convulsive status epilepticus/decompensation)
*Fisher exact test p-value
